# Spring warming increases the abundance of an invasive specialist insect: links to phenology and life history

**DOI:** 10.1038/s41598-017-14989-3

**Published:** 2017-11-01

**Authors:** Rui-Ting Ju, Lei Gao, Shu-Juan Wei, Bo Li

**Affiliations:** 1 Ministry of Education Key Laboratory for Biodiversity Science and Ecological Engineering, Fudan University, Shanghai, 200438 China; 2 Institute of Plant Protection, Shanghai Academy of Landscape Architecture Science and Planning, Shanghai, 200232 China

## Abstract

Under global warming, shifts in phenological synchrony between insects and host plants (i.e., changes in the relative timing of the interaction) may reduce resource availability to specialist insects. Some specialists, however, can flexibly track the shifts in host-plant phenology, allowing them to obtain sufficient resources and therefore to benefit from rising temperatures. Here, we investigated the effects of experimental warming on the life history of an invasive, specialist lace bug (*Corythucha ciliata*) and on the leaf expansion of its host plant (*Platanus* × *acerifolia*) in two spring seasons under field conditions in Shanghai, China. We found that a 2 °C increase in mean air temperature advanced the timing of the expansion of host leaves and of the activities of overwintering adult insects in both years but did not disrupt their synchrony. Warming also directly increased the reproduction of overwintering adults and enhanced the development and survival of their offspring. These results indicate that *C. ciliata* can well track the earlier emergence of available resources in response to springtime warming. Such plasticity, combined with the direct effects of rising temperatures, may increase the insect’s population size and outbreak potential in eastern China under climate warming.

## Introduction

Global warming is likely to substantially affect population dynamics and species interactions, making it challenging to predict climatic effects on species across taxa and trophic levels^1–6^. For herbivorous insects, the effects of climate warming can be direct, through changes in herbivore physiology, or indirect, through changes in herbivore interactions with other organisms^7–9^. In terms of species interaction, herbivores interact with and depend on host plants, and the long-term population sizes of herbivorous insects under warming conditions will therefore be greatly influenced by the availability of host-plant resources^10^. Given that non-native invasive insect herbivores have major impacts on ecosystem function and agricultural production^11,12^, it has been a focal issue about how invasive insects respond to climate warming in recent years^7^. While some studies have shown that climate warming may increase the prevalence of alien invasive insects^13,14^, those studies mainly concern the direct effects of rising temperatures on the removal of the physiological constraints of the invasive insects^4^; the indirect effects of resource availability on non-native insects have been less studied^7^.

The qualities and quantities of food resources used by herbivorous insects are often associated with the phenologies of their host plants^15,16^. For specialist insects in particular, variation in plant phenologies may result in food shortage and may thus affect insect development, survival, and reproduction^17^. Understanding such variation is important for predicting and controlling plant damage by insect pests^18^. Because of climatic warming, many deciduous tree species have advanced their spring phenologies (e.g., they exhibit early leaf expansion and flowering)^19,20^. Similarly, many herbivorous insects have begun to emerge earlier as temperatures have increased^15,21^. In this case, the degree to which the advancement in specialist-insect phenology is synchronous with the advancement in host-plant phenology will affect the extent of insect outbreaks and plant damage in the warming future^18^.

Because of species-specific sensitivity to variations in temperature, climate change might lead to phenological mismatches (i.e., changes in the relative timing of the interaction) between species that interact across trophic levels^15,16,22,23^. Especially in warmer springs, development of overwintering insects seems to be changing at a steeper rate than that of their host plants, which is likely to result in insects appearing while host plants are still dormant^24^. Such phenological decoupling may reduce the population fitness of specialist herbivorous insects and may thus cause a decline in their abundance under climate warming^15,25^. Some specialist insects, however, may maintain phenological synchrony with host plants through behavioral or physiological plasticity (e.g., flexibility in activity, foraging, development, and reproduction) as well as rapid genetic changes in response to the changing environment^26–28^. These insects, therefore, may still obtain sufficient food resources under climate warming^29,30^.

Although most invasive insects are generalist species, a few invaders are specialist herbivores (e.g., *Acanthoscelides pallidipennis* (Coleoptera: Bruchidae), *Viteus vitifoliae* (Homoptera: Phylloxeridae))^31^. Given that climate warming is advancing the phenologies of many host plants, the population density of some non-native, specialist insects may depend on their plasticity associated with phenologies^18^. If these insects can adapt to variation in the timing of host-plant phenologies at local scales, we can then expect that they may benefit from the earlier emergence of resources under climate warming i.e., that they may benefit indirectly from climate warming. If rising temperatures promote their development and reproduction, these insects may also benefit directly from climate warming^4^. To date, however, empirical studies simultaneously investigating such direct and indirect effects on invasive, specialist insect species remain rare.

Here, we investigated the effects of simulated warming on the phenologies of an invasive specialist, the lace bug *Corythucha ciliata* (Say) (Hemiptera: Tingidae), and its host plant, the London plane tree *Platanus* × *acerifolia* (Platanaceae), in Shanghai, China. We also examined how warming affects the insect’s development, survival, and reproduction. Although the long-distance spread of *C. ciliata* results from human activity and wind dispersal, host resources and climate change will affect outbreaks of this invasive bug in local areas^14,32^. In Shanghai, host resources of *C. ciliata* are most available soon after *P*. × *acerifolia* leaf expansion in spring because young leaves provide suitable food and habitat for the adults^32^. Hence, the synchronous appearance of the adults and the young leaves in early spring greatly affects insect population dynamics in subsequent seasons. We tested the hypotheses that *C. ciliata* can flexibly track the timing of leaf expansion of its host plant in warming springs, and that warming springs can increase the abundance of this specialist insect in Shanghai.

## Results

### Phenologies of leaves and overwintering adults

**Figure 1:**
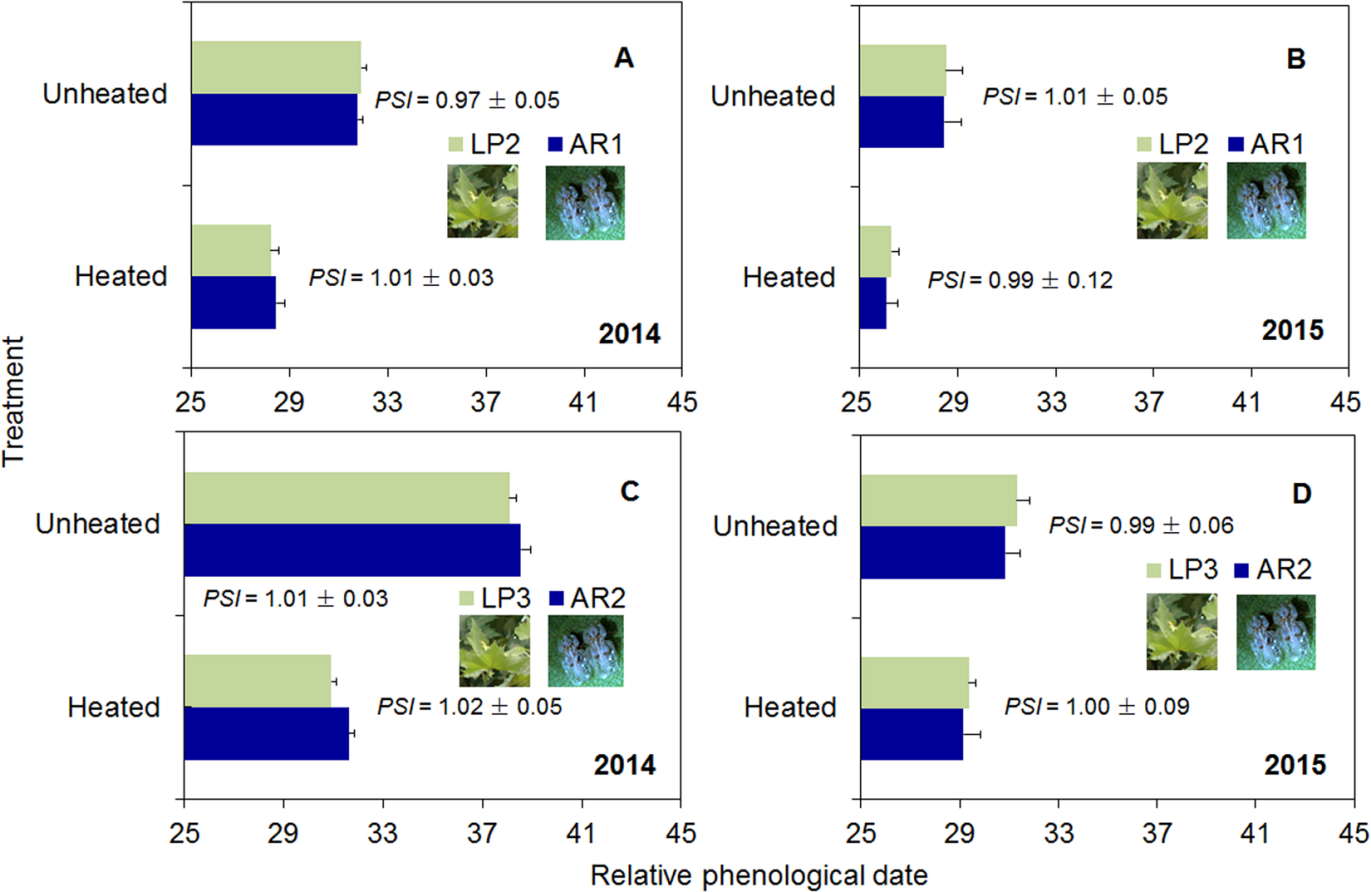
Phenologies of *C. ciliata* overwintering adults (AR1 and AR2) and of *P*. ×*acerifolia* leaves (LP2 and LP3) in heated or unheated field plots in the springs of 2014 (**A**,**C**) and 2015 (**B**,**D**). AR1: Cumulative 1–10% adults revived; AR2: Cumulative 11–50% adults revived; LP2: Cumulative 10–50% leaves fully expanded; LP3: Cumulative 51–100% leaves fully expanded. PSI (the phenological synchrony index) indicates the degree of synchrony between each pair of phenologies (either AR1/LP2 or AR2/LP3). PSI was calculated as the ratio of the relative phenophases of overwintering adults (the number of days after 1 March when AR1 or AR2 occurred) and plant leaves (the number of days after 1 March when LP2 or LP3 occurred). Values are means + SE. Statistical results are provided in Table 1.

Although experimental warming significantly advanced the timing of both overwintering adult revival (AR1 and AR2) and leaf emergence (LP2 and LP3) in 2014 and 2015, warming did not disrupt the phenological synchrony (PSIs ~ 1) between the adults and the leaves (AR1/LP2 and AR2/LP3) in either year (Fig. 1, Table 1). Warming advanced the phenophases by 3–4 days for AR1/LP2 (Fig. 1A,B) and by 2–7 days for AR2/LP3 (Fig. 1C,D). Moreover, the phenologies of adults and plant leaves were significantly earlier in 2015 than in 2014 under the same warming treatment **(**Fig. 1, Table 1).

**Table 1:** General linear and linear regression models (GLMs and LRMs) testing the effects of year, warming treatment (warm), and their interaction on the phenologies of *C. ciliata* overwintering adults and *P*. × *acerifolia* leaves, and on the life-history traits of the overwintering adults and their progeny.

Category	Response variable	Source of variation	*df*	*F*-value	*P*-value
Phenologies	AR1	Year	1, 48	**17.53**	**<0.001**
		Warm	1, 48	**40.33**	**<0.001**
		Year × Warm	1, 48	0.04	0.835
	AR2	Year	1, 48	**28.25**	**<0.001**
		Warm	1, 48	**47.11**	**<0.001**
		Year × Warm	1, 48	**18.47**	**<0.001**
	LP2	Year	1, 48	**19.25**	**<0.001**
		Warm	1, 48	**52.28**	**<0.001**
		Year × Warm	1, 48	**5.82**	**0.02**
	LP3	Year	1, 48	**23.94**	**<0.001**
		Warm	1, 48	**75.63**	**<0.001**
		Year × Warm	1, 48	**46.36**	**<0.001**
	PSI of AR1/LP2	Year	1, 48	0.07	0.795
		Warm	1, 48	0.17	0.685
		Year × Warm	1, 48	2.73	0.106
	PSI of AR2/LP3	Year	1, 48	1.84	0.182
		Warm	1, 48	0.07	0.795
		Year × Warm	1, 48	0.04	0.852
Longevity and fecundity of overwintering adults	Longevity	Year	1, 48	0.03	0.855
		Warm	1, 48	**6.32**	**0.016**
		Year × Warm	1, 48	3.18	0.081
	Oviposition period	Year	1, 48	**64.07**	**<0.001**
		Warm	1, 48	0.17	0.678
		Year × Warm	1, 48	0.26	0.610
	Fecundity	Year	1, 48	1.90	0.175
		Warm	1, 48	**14.26**	**<0.001**
		Year × Warm	1, 48	0.30	0.587
Demography of overwintering adults	Gross fecundity rate	Year	1, 4	0.72	0.486
		Warm	1, 4	**187.68**	**0.045**
	Net fecundity rate	Year	1, 4	2.30	0.268
		Warm	1, 4	6.32	0.241
	Daily egg production	Year	1, 4	1.32	0.369
		Warm	1, 4	1.19	0.472
Life-history traits of progeny	Egg hatch	Year	1, 48	0.11	0.742
		Warm	1, 48	**8.45**	**0.006**
		Year × Warm	1, 48	0.18	0.677
	Developmental time of eggs	Year	1, 48	2.02	0.162
		Warm	1, 48	**76.08**	**<0.001**
		Year × Warm	1, 48	0.26	0.616
	Nymph survival	Year	1, 48	0.70	0.407
		Warm	1, 48	**9.40**	**0.004**
		Year × Warm	1, 48	0.02	0.888
	Developmental time of nymphs	Year	1, 48	0.14	0.706
		Warm	1, 48	**42.45**	**<0.001**
		Year × Warm	1, 48	0.53	0.472
	Female percentage	Year	1, 48	**47.00**	**<0.001**
		Warm	1, 48	0.24	0.623
		Year × Warm	1, 48	0.10	0.758

Year: 2014 and 2015. Warming treatment: heated and unheated. Phenological abbreviations (AR1, AR2, LP2, and LP3) are explained in Table 4. PSI is the phenological synchrony index that indicates the degree of synchrony between each pair of phenologies (either AR1/LP2 or AR2/LP3). PSI was calculated as the ratio of the relative phenophases of overwintering adults (the number of days after 1 March when AR1 or AR2 occurred) and plant leaves (the number of days after 1 March when LP2 or LP3 occurred). Significant results are shown in bold (*P* < 0.05).

### Longevity and fecundity of overwintering adults

**Figure 2:**
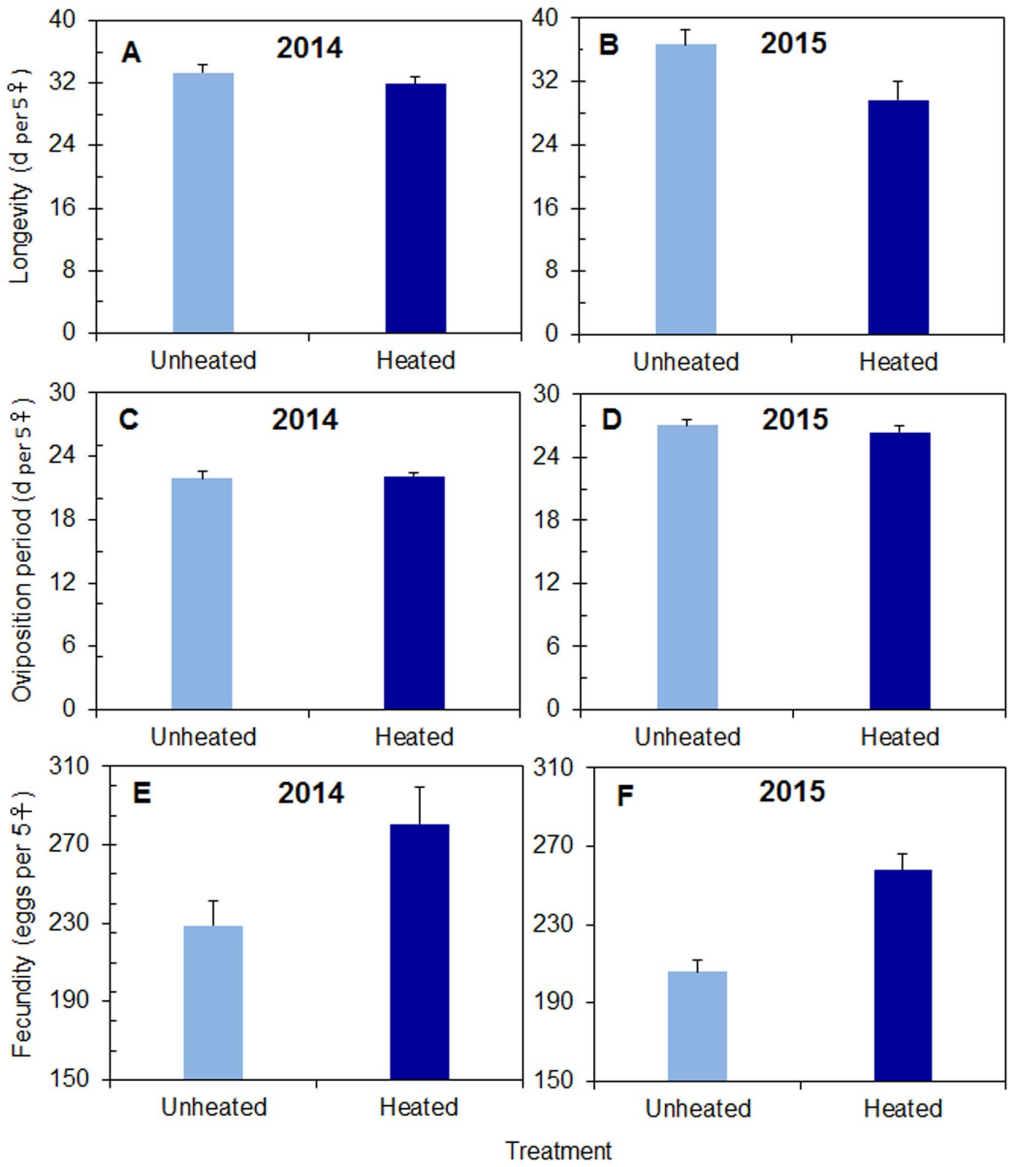
Longevity (**A**,**B**) and fecundity (**C**–**F**) of overwintering adult females of *C. ciliata* in cages with *P*. × *acerifolia* leaves. The cages were located in heated or unheated field plots in the springs of 2014 (**A**,**C**,**E**) and 2015 (**B**,**D**,**F**). Values are means + SE and were obtained with each group of five females. Statistical results are provided in Table 1.

**Figure 3:**
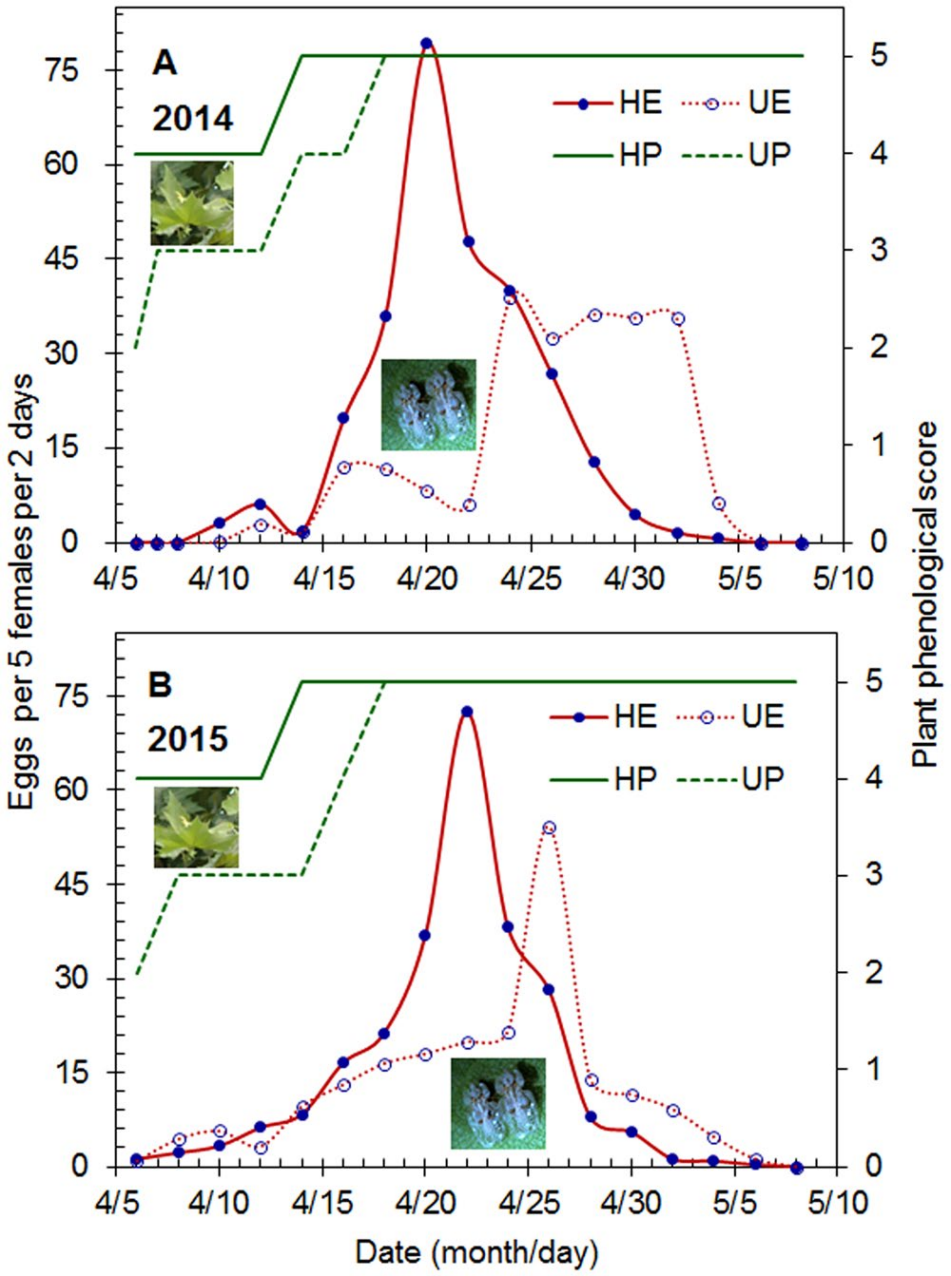
Number of eggs per 5 *C. ciliata* females per 2 days (*M*x) oviposited after adults revived from overwintering and the corresponding plant phenology scores in heated or unheated field plots in the springs of 2014 (**A**) and 2015 (**B**). HE and UE refer to the numbers of eggs produced in the heated and unheated treatment, respectively. HP and UP refer to the plant phenological scores in the heated and unheated treatment, respectively. Plant phenological scores are explained in Table 4.

For the reviving overwintering adults, experimental warming significantly decreased the longevity of female adults in both 2014 and 2015 (Fig. 2A,B, Table 1). Warming did not affect the oviposition period of overwintering female adults (Fig. 2C,D, Table 1), but it advanced the peak oviposition date by 4 days in both years (Fig. 3). When the peak oviposition occurred, the leaf phenology score was 5 (91–100% of full leaf expansion) (Fig. 3). The mean number of eggs laid by per 5 overwintering females was significantly higher in the heated than in the unheated treatment in both years (Fig. 2E,F, Table 1). When demographic parameters were analyzed, warming increased the gross fecundity rate in both years but did not affect the net fecundity rate or daily egg production in either year (Tables 1 and 2).

**Table 2:** Demographic parameters of overwintering adult females of *C. ciliata* in heated and unheated cages kept outdoors in the springs of 2014 and 2015.

Year	Treatment	Gross fecundity rate per five females	Net fecundity rate per five females	Daily egg production per five females
2014	Heated	280.0	278.9	9.2
	Unheated	228.1	220.6	6.8
2015	Heated	247.5	214.9	7.2
	Unheated	202.5	193.9	5.3

Demographic parameters were obtained from groups of five females. These parameters were estimated as described in the section of Data analysis. Statistical results are provided in Table 1.

### Development and survival of the progeny of overwintering adults

For the progeny of overwintering adults, experimental warming significantly increased egg hatch, nymph survival, and their developmental rates in both 2014 and 2015 (Tables 1 and 3). Of the nymph progeny that developed into adults, the female percentages did not differ between heated and unheated treatments, although the percentages were higher in 2014 than in 2015 (Tables 1 and 3).

**Table 3:** Life-history traits of progeny of overwintering *C. ciliata* adults in heated and unheated cages kept outdoors in the springs of 2014 and 2015.

Year	Treatment	Egg hatch (%)	Developmental time of eggs (d)	Nymph survival (%)	Developmental time of nymphs (d)	Female percentage (%)
2014	Heated	90.5 ± 1.0	15.0 ± 0.1	67.9 ± 3.3	17.1 ± 0.3	74.0 ± 5.1
	Unheated	87.1 ± 1.1	17.8 ± 0.1	56.2 ± 2.4	21.9 ± 0.3	72.0 ± 5.6
2015	Heated	90.9 ± 1.2	14.0 ± 0.5	71.0 ± 3.8	16.8 ± 0.1	45.4 ± 1.1
	Unheated	86.9 ± 1.3	17.0 ± 0.2	66.5 ± 4.2	23.4 ± 2.0	45.9 ± 2.4

Values are means ± SE. Statistical results are provided in Table 1.

## Discussion

Variations in phenology can greatly affect the interactions between insects and their host plants^33^. Because of species-specific variation in response to climate, recent spring warming has changed the phenological synchrony between many but not all insect herbivores/pollinators and their host plants^34–39^. Although a large number of studies have discussed the potentially decreased fitness of insect herbivores caused by phenological decoupling^17,24,25,33^, only a few studies have quantified the fitness or demographic effects of phenological synchrony on insects^16,40^. Through experimental warming, our study generated three main findings at a local scale. First, experimental warming advanced the phenologies of both *C. ciliata* (an invasive, specialist herbivorous insect) and its host plant *P*. × *acerifolia* (a native deciduous tree) in spring in Shanghai. Second, warming did not alter the temporal synchrony between overwintering adult revival and leaf emergence. Third, warming increased the reproduction of overwintering adults and enhanced the development and survival of their offspring in spring. Because of both the direct effects of warming on life history and its indirect effects on trophic coupling, the increases in temperature may increase the insect’s abundance under springtime warming in Shanghai.

Previous studies have often used the date of first appearance of an event (e.g., the first animal seen in spring, the first plant flowering in summer) to estimate changes in phenology, but this is unlikely to reasonably represent the whole population^41,42^. The cumulative responses of individuals rather than the first appearances of events are likely to provide better estimates of phenologies of herbivores and their host plants^43^. In this study, we used the percentage of cumulative changes in individual response to describe a certain phenology at the population level and to thereby reduce the potential bias introduced by basing phenology on the behavior of only a few individuals. On the other hand, we used experimental warming in outdoor, open-air macrocosms (pots in cages) to simulate a higher temperature profile (~2 °C) predicted in the future. This method produced the critical mean increase in temperature under global warming estimated by IPCC^6^ and should be useful for assessing the shifts in phenologies of insects and their host plants caused by climate warming at a local scale. Previous studies have shown that, relative to experimental warming, model simulations may provide more dynamic predictions of how global warming will change phenologies at a large scale^44,45^. In particular, simulated data of the performance of a species across its entire range could be more useful than a pot experiment carried out over only a few years at a single site.

At the global scale, we can draw two general conclusions based on most previous reports concerning phenology and climate warming. First, there is a very clear shift in phenology towards an earlier date due to climate warming^34–39^. Second, species from different taxonomic groups shift at different rates^15,16,22,23^. Because increased temperatures may advance phenologies to different degrees among species that interact in space and time, trophic decoupling is possible for herbivores. At a local scale, however, we found that although a 2 °C increase in mean air temperature significantly advanced the timing of *C. ciliata* revival and leaf emergence, their phenological synchrony was not disrupted. Maintenance of phenological synchrony has also been found in several other species interacting over trophic levels^38,39^. From these cases, we suggest that phenological asynchrony between insects and their host plants may not be a universal phenomenon under climate warming.

While the phenologies of *C. ciliata* adults and *P*. × *acerifolia* leaves were relatively synchronous in each year of this study, adult revival and leaf emergence in heated and unheated plots occurred earlier in 2015 than in 2014. This phenomenon coincided with air temperatures in late March, which were higher in 2015 than in 2014 in both kinds of plots. This suggests that air temperatures in late March may be useful for predicting the spring phenologies of *C. ciliata* adults and *P*. × *acerifolia* leaves. In addition to being correlated with warm temperatures in spring, *P*. × *acerifolia* leaf expansion is also correlated with cold temperatures in winter^45,46^. The effects of low temperatures before spring were not examined in this study. Moreover, a past study has suggested that changes in temperature may affect the sex ratio of insects^47^. In terms of the sex ratios of the overwintering-adult progeny in the current study, the female percentages were significantly higher in 2014 than in 2015, which may also be correlated with different spring temperatures between the years.

Under climate-change scenarios, differences in the physiological responses to climatic fluctuations between organisms have explained phenological decoupling^15,25^, but those factors responsible for a tight synchrony of insect herbivores with their host plants are unclear^36^. We suspect that a tight coupling in phenologies may result from the co-evolution between insects and host plants, and from insect plasticity. During their co-evolution, an insect species and its host plant have optimized their phenological relationship in terms of chronological vs. physiological time. Additionally, despite the existence of mutual pressure and response in co-evolution, it is likely that host-plant phenology leads to a directional selection on herbivore phenology^15^. Although this selection pressure will vary between years even in the absence of climate change, the selection pressure is likely to be substantially greater under climate change. Insects, however, can utilize their phenotypic plasticity (e.g., high tolerance of starvation, diversified feeding and reproductive performances) to adapt to changes in selection intensity^4,48–50^. In this study, the tight phenological synchrony of *C. ciliata* adults with their host plant may be associated with a high level of tolerance of temperature changes in the adults. That is to say, by tolerating an increased temperature of 2 °C, the adults can synchronize their activities with leaf expansion so that plant resources match the insect’s requirements in spring. Although spring warming means faster accumulation of physiological time units for both plants and insects^7^, when temperatures rise but plant leaves are unavailable, the adults may be able to remain dormant until leaves expand. Such phenotypic plasticity may help explain why *C. ciliata* maintains phenological synchrony with *P*. × *acerifolia* under different thermal conditions.

Different species are likely to have evolved different responses to variations in their food resources^51^, and the role of phenological synchrony may differ depending on herbivore feeding habit^15^. Specifically, this synchrony is more important for specialist insects than for generalists^15^. The explanation is simple in that when phenological asynchrony occurs, generalist herbivores are able to use a variety of other host resources^15,35^. Specialists, however, have only a limited diet and must adapt to changes in the host resources through behavioral or physiological plasticity. Otherwise, they could become extinct in a changing environment. As a specialist insect herbivore, *C. ciliata* has become more prevalent under climate warming in Shanghai^14,52^. Apart from climatic conditions, the prevalence of *C. ciliata* depends on the availability of *P*. × *acerifolia*. *C. ciliata* is an oligophagous insect specialized on *Platanus* spp. (*P. occidentalis*, *P*. × *acerifolia*, and *P. orientalis*). In Shanghai, *P*. × *acerifolia* is very common but *P. occidentalis* and *P. orientalis* are not. Therefore, *C. ciliata* must rely on *P*. × *acerifolia* leaves for food and shelter and must synchronize its activity with *P*. × *acerifolia* leaf expansion in spring. Evidence suggests that the ability of an insect to adapt to changes in host resource is heritable^50,53,54^. We think that *C. ciliata* may maintain its tight phenological coupling with its host plant in the warming future. Our study, however, only included a 2 °C increase in mean air temperature, which appears to be well within the thermal safety margins of *C. ciliata* and *P*. × *acerifolia* ^14,52^. Given uncertainty about the level of global warming, there may be warmer temperatures that suppress the development and growth of the plant, the herbivore, or both. This may uncouple the synchrony between the two species and needs further estimation.

Under climate change, phenological synchrony and rising temperatures have important ecological consequences^55,56^. For insect herbivores, these consequences have been assessed by a few studies that related resource availability and life history responses. For example, warming-induced phenological shifts alter the timing of ecological interactions between the forest tent caterpillar (*Malacosoma disstria* (Lepidoptera: Lasiocampidae)) and its host trees, which alters development time of the caterpillar and therefore affects its outbreak dynamics in northern Minnesota, USA^36^. Phenological mismatch between the western tent caterpillar (*Malacosoma californicum pluviale* (Lepidoptera: Lasiocampidae)) and its host plant (*Alnus rubra* (Betulaceae)), in contrast, has no net effect on the caterpillar because the larvae can accelerate development once leaves are available^16^. In our study, experimental warming caused advances in the leaf expansion of *P*. × *acerifolia* and synchronous advances in the activity of *C. ciliata*. By providing resources to the insect at an earlier time, warming also advanced the oviposition date and enhanced the fecundity of overwintering adults. In addition, the progeny of the overwintering adults benefitted from the warming due to increased survival and accelerated development. Warming is particularly important for the eggs of *C. ciliata* because exposure of the eggs to cooling conditions for a prolonged period can significantly affect their hatches and development in spring^57^. Our results provide additional empirical evidence of the positive effects of climate warming on insect herbivores related to phenological synchrony and life history. As indicated in our previous studies, the life-history traits of *C. ciliata* can also benefit from other climate-change patterns, such as increases in mean temperature and heat waves in summer^14,52,57–59^. Together, these results suggest that climate warming may increase the number of generations per year and potentially favor population outbreaks of *C. ciliata* over large regions. We infer that this insect herbivore may continue to expand its invasive ranges at the global scale due to the wide distribution of its host plants.

Our results with *C. ciliata* may contribute to predictions of how climate warming will affect similar specialist species. Recently, climate warming has led to increases in leaf biomass, i.e., climate warming has indirectly benefitted herbivorous insects by increasing food resources^60^. If specialist herbivorous insects are able to match the timing of their behavior or physiology with the changes in the timing of host resource availability, they may benefit rather than suffer from the phenological changes caused by climate warming. These herbivorous insects may also benefit from the direct effects of elevated temperatures on their development and reproduction^4^. Given that at least some specialist insects can flexibly track the shifts in host resources, we predict that warming springs may increase population sizes of such specialists and may increase their tendency to outbreak. Such information is useful for improving risk assessments and management strategies for these insect herbivores. Further studies are needed, however, to answer the following questions: (i) If both temperature and plant metabolites affect the phenology of *C. ciliata*, how are overwintering adults able to remain dormant when temperatures rise but when the host plant is absent or still dormant? (ii) Do the responses of invasive insect species to ambient and elevated temperatures differ between their native and invasive ranges?

## Materials and Methods

### Study site

**Figure 4:**
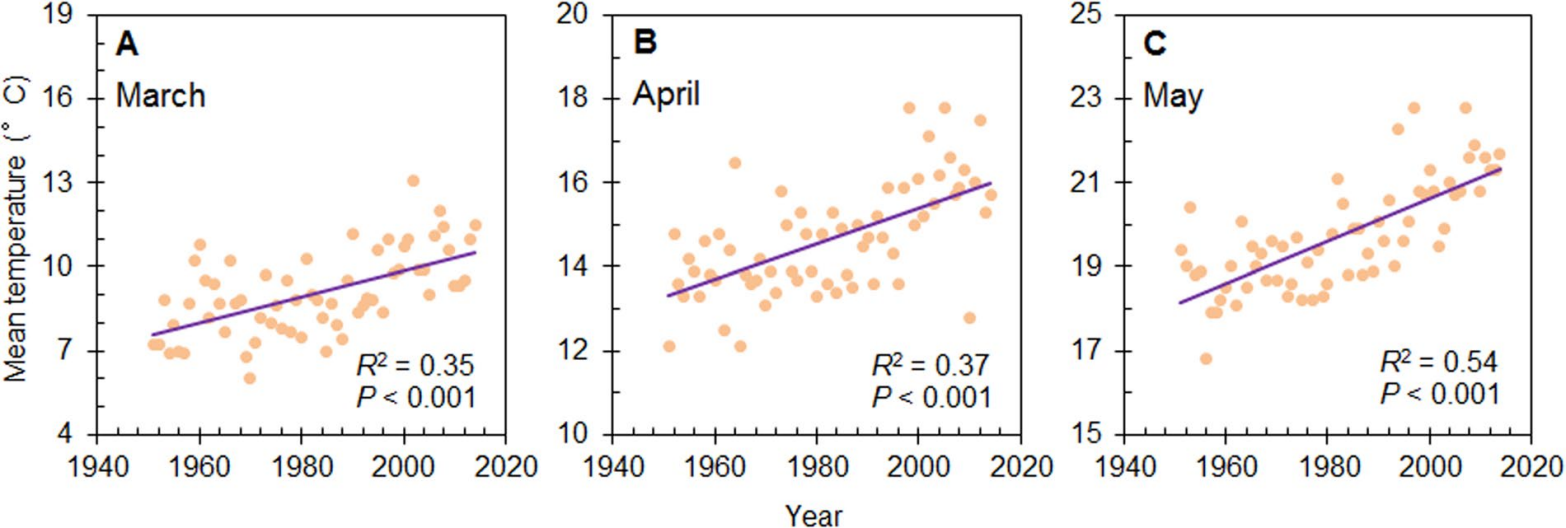
Mean temperatures in March (**A**), April (**B**), and May (**C**) in Shanghai from 1951 to 2014. Data were obtained from the China Meteorological Data Sharing Service System (http://cdc.cma.gov.cn). The weather stations providing these data are located in Longhua, Shanghai (31.10 °N, 121.26 °E).

Our experiment was conducted outdoors with natural photoperiod and humidity at the Shanghai Academy of Landscape Architecture Science and Planning (31°09′ N, 121°26′ E, 7.01 m a.s.l.), China. Shanghai is in a subtropical moist monsoon climatic zone. The region has four distinct seasons, i.e., spring (March to May), summer (June to August), autumn (September to November), and winter (December to February). At the experimental site, mean temperatures in spring have steadily risen over the past 63 years (1951–2014) (Fig. 4). The means in the most recent 10-year period were 2–3 °C warmer than those in the 1950’s.

### Study system

*C. ciliata* and *Platanus* trees are amenable species for studying the effects of climate warming on invasive specialist insects and on herbivore-host interactions^61^. Native to North America, *C. ciliata* is an invasive insect in Europe, Australia, South America, and East Asia^32^. In China, this invasive species was first found in Changsha, Hunan Province in 2002^62^, and then spread rapidly to 11 other provinces between latitudes 26 °N and 37 °N^32,58^, where Shanghai is a typical invaded region. As noted in the Discussion, *C. ciliata* is a specialist herbivore that feeds only on sycamore trees, including *P. occidentalis*, *P. orientalis*, and *P*. × *acerifolia* ^32^. These are deciduous species and have similar phenological characteristics in Shanghai (personal observation by the first author). Nymphs and adults of *C. ciliata* pierce and suck juices of *Platanus* leaves, resulting in stunted growth and sometimes in tree death^32^. In terms of resource availability, *P*. × *acerifolia* is the main host for *C. ciliata* in Shanghai because it is the main tree planted along streets and is also widely distributed in the field. Although *P. occidentalis* and *P. orientalis* are also the potential hosts of *C. ciliata*, they are much less abundant than *P*. × *acerifolia* and are mainly found in botanical gardens. The prevalence of *C. ciliata* in Shanghai, therefore, depends mainly on its interaction with *P*. × *acerifolia*.

Because of a lack of effective enemies, *C. ciliata* has become a dominant herbivore on *P*. × *acerifolia* in China. *C. ciliata* has a short life cycle and five generations per year in Shanghai^57^. In late October, the adults of *C. ciliata* overwinter under the exfoliating outer bark of the host tree or in the dry branches and fallen leaves around the tree. The adults become active when the average daily temperature is about 15 °C in spring^32^. After reviving, the adults require host leaves for food and for reproductive habitat. If they become active before host leaves expand, the adults may not experience maximum resource availability, with obvious detrimental consequences for *C. ciliata* population. Following recent climate warming, we found that the yearly abundance of *C. ciliata* was continuously increasing on *P*. × *acerifolia* in late spring. This suggests that trophic decoupling is probably not occurring in this plant-herbivore interaction, and that *C. ciliata* populations may even be enhanced by warmer temperatures in Shanghai.

### Plant culture and insect collection

Seedlings of *P*. × *acerifolia* (diameter of middle part of trunk = 3 cm, height = 100–110 cm, number of buds ≥ 10) were purchased in Jiangsu Province, which has a climate similar to that of Shanghai. In December 2013 and 2014, each *P*. × *acerifolia* seedling was planted in a pot (diameter = 18 cm, height = 14 cm) containing soil (soil depth in the pot = 10 cm). Before planting, the soil had been treated with a fungicide (carbendazole) to prevent pathogen infections. The soil surface in each pot was covered with a 5-cm-thick layer of dry *P*. × *acerifolia* bark. In the middle of February of 2014 and 2015, each seedling was pruned to a height of 100 cm and had 9–10 living buds remaining. Overwintering adults of *C. ciliata* were collected from *P*. × *acerifolia* on 25 February 2014 and on 26 February 2015 along the Caobao Road (31°09′34″ N, 121°22′27″ E) in Shanghai. The adults were kept in a dark box at collection time and within 12 h were transferred onto the bark in pots (200 adults per pot). Each pot was immediately transferred to a cage (length = 50 cm, width = 50 cm, height = 130 cm; one pot per cage). The cage was made of 40-mesh (sieve size = 0.425 mm) white nylon screen that prevented insect escape. The screening reduced solar radiation by 30% but altered temperature only slightly.

### Experimental warming

**Figure 5:**
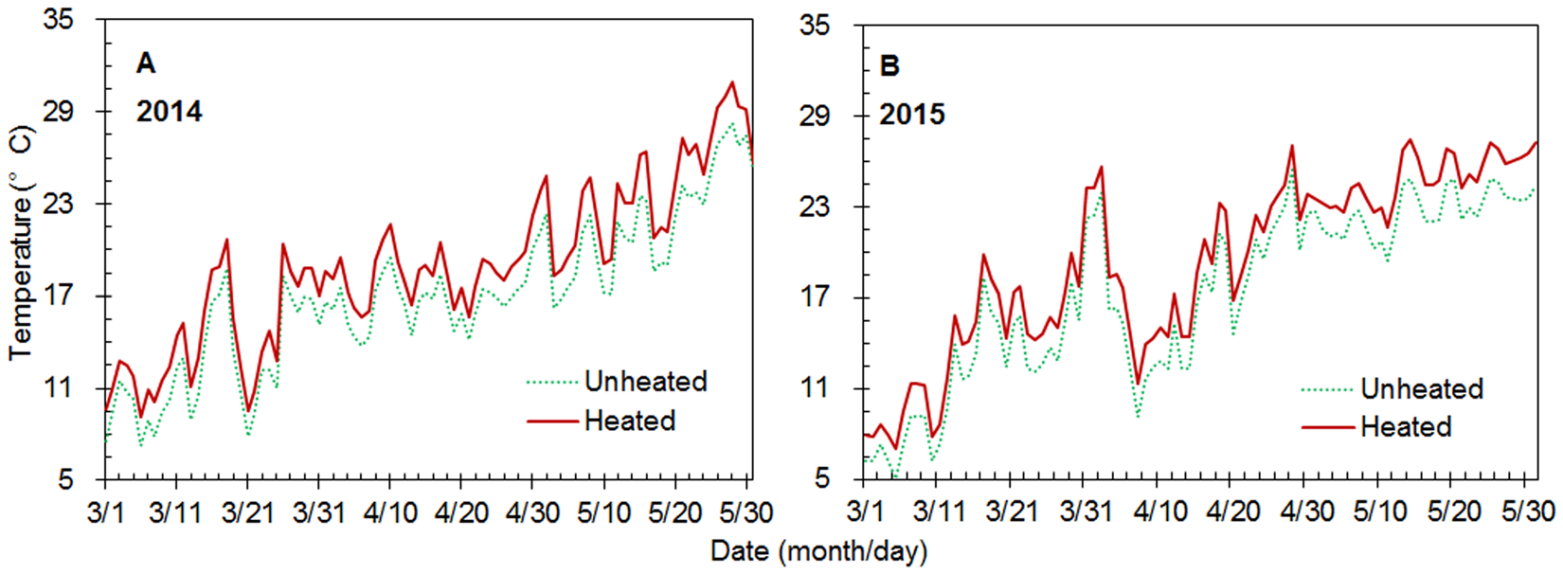
Daily air temperatures in heated and unheated plots from March to May in 2014 (**A**) and 2015 (**B**). Air temperature was measured with a probe that was located about 50 cm above the soil surface of each experimental pot.

The day that pots with seedlings and overwintering adults were placed in cages was recorded as the start date of the experiment in each year. There were 24 cages, and these were placed in 12 pairs of 2 m × 4 m field plots at the study site, with one cage per plot. The plots were arranged in pairs, and the edges of adjacent plots were 2 m apart. Each cage containing one seedling in a pot and 200 *C. ciliata* overwintering adults was placed at the center of each plot. In each pair of outdoor plots, one cage was subjected to a warming treatment (heated) and the other was kept at ambient temperature (unheated control). The warming treatment was achieved by suspending a 165 cm × 15 cm MSR-2420 infrared radiator (Kalglo Electronics, Bethlehem, PA, USA) above the cage. The height from the bottom edge of the radiator to the soil surface was 1.5 m. ‘Dummy’ heaters with the same shape, size, and installation as the real heaters were suspended over each control cage to mimic the shading effects of the real heater. In each cage, air temperature was measured with a JK–32U multiplex automatic temperature recorder (Ailian Electronics, Changzhou, Jiangsu, China). The thermometer probe was located 50 cm above the soil surface. Data were recorded hourly from March 1 to May 31 in 2014 and 2015. Compared to unheated control, experimental warming increased the mean air temperature in springtime by 2.02 ± 0.11 °C in 2014 and by 2.01 ± 0.13 °C in 2015 (Fig. 5). As noted earlier, photoperiod and relative humidity were not experimentally modified or measured.

### Phenologies of leaves and overwintering adults

Beginning on 1 March in 2014 and again in 2015, expansion of *P*. × *acerifolia* leaves and revival of *C. ciliata* overwintering adults were assessed daily at 15:30–16:00 using a phenological scoring system (Table 4). This was done until late April in each year. Adults were considered revived if they were active and walked away from their overwintering places in the cage. According to our previous long-term observations in the field in Shanghai^63^, when *C. ciliata* overwintering adults began to emerge (referred to as “cumulative 10% adults reviving” and abbreviated AR1), *P*. × *acerifolia* was at the phenological stage of “young leaves protruding partly from buds” (abbreviated LP2); when the adult emergence peaked (referred to as “cumulative 50% adults reviving” and abbreviated AR2), the plant phenological stage was “10–50% of full leaf expansion” (abbreviated LP3). We therefore used the paired phenologies AR1/LP2 and AR2/LP3 to analyze the phenological synchrony between *C. ciliata* and its host plant. We used the relative phenophase to indicate the unit of phenological variables. In each year, the number of days after 1 March when a phenological event occurred was recorded as its relative phenophase. Each treatment (heated or unheated) was represented by 12 replicate cages (described in the ‘experimental warming’ section), with one seedling and 200 randomly selected adults per replicate cage. By the end of phenological tests, almost all of the adults had revived. The reviving adults were collected and used to determine the longevity and fecundity of overwintering adults (as described in the next section).

**Table 4:** Phenological scoring of *C. ciliata* overwintering adults and of *P*. × *acerifolia* leaves in spring.

Category	Phenology	Score	Abbreviation
Overwintering adults	0 adults revived	0	AR0
	Cumulative 1–10% adults revived	1	AR1
	Cumulative 11–50% adults revived	2	AR2
	Cumulative 51–100% adults revived	3	AR3
Plant leaves	All buds fully closed	0	LP0
	Swollen buds just opened	1	LP1
	Young leaves partly protrude from buds	2	LP2
	Cumulative 10–50% leaves fully expanded	3	LP3
	Cumulative 51–90% leaves fully expanded	4	LP4
	Cumulative 91–100% leaves fully expanded	5	LP5

### Longevity and fecundity of overwintering adults

A reproduction experiment was also conducted under outdoor conditions. When virginal adults emerged from the overwintering stage that had been maintained in the heated or unheated cages, they were paired (one male and one female) using a small, fine brush; this was done on 3 April in 2014 and in 2015. One group of five pairs was placed on a young leaf on a branch that was cut from *P*. × *acerifolia* trees that had been planted in a greenhouse in December 2013. The branch, whose base was inserted in a water-filled bottle, and the associated group of five pairs were placed in a small cage (length = 23 cm, width = 23 cm, height = 54 cm) covered with 40-mesh white nylon screen. The leaf provided food and oviposition sites for the adults. Each of the 24 small cages was placed adjacent to one of the 24 large cages used for the phenological observations described in the previous section. This allowed us to continue the experimental conditions (heated or unheated as described in the previous section) in the reproduction experiment. Because the leaf and thermometer were at the same approximate height, the temperatures recorded were similar those experienced by the individual adults. Adult survival and reproduction were assessed daily. When oviposition occurred, leaves with attached eggs were removed, and another branch with new leaves was supplied for oviposition until all five females died. The eggs deposited on leaves were counted with a binocular stereoscope (MZ 16 A, Leica Microsystems Ltd., Wetzlar, Germany). As described in our previous reports^14,52^, the following data for each paired group were collected: length of oviposition period, female longevity, and fecundity. Each treatment (heated and control) was represented by 12 replicate cages, with five pairs of adults in each cage.

### Development and survival of the progeny of overwintering adults

The newly deposited eggs on the leaves (i.e., eggs of the same age in days) and associated branch that were obtained as described in the previous section were subjected to the same outdoor conditions (heated or unheated) as their parents. The eggs were examined daily until no nymphs hatched after 7 successive days. Hatching rate and the mean time required for development into nymphs in each cage were determined. After nymphs emerged, they were reared on *P*. × *acerifolia* leaves as described in the previous section. The nymphs were examined daily, and survival and the mean time required for development into adults in each cage were assessed. Leaves were replaced every 2 days. After eclosion of all adults, the sex ratio in each cage was determined. Each treatment (heated and control) was represented by 12 replicate cages, with ≥ 30 eggs and 30 nymphs per cage.

### Data analysis

General linear models (GLMs) or linear regression models (LRMs) were used to test the effects of year, experimental warming, and their interaction on the phenologies of overwintering adults and host leaves, and the life-history traits (survival, reproduction, and development time) of the adults and their progeny. The models included year, warming treatment, and their interaction as fixed effects. The count data were fitted to GLMs by using the family of Quasi-Poisson. The ratio or percentage data were fitted to LRMs after natural logarithm transformation. All analyses were performed in R version 3.3.3 with a significance level of alpha = 0.05^64^.

A phenological synchrony index (PSI) was used to assess the synchrony of each paired phenologies (AR1/LP2, AR2/LP3). This index was evaluated as follows:

1 $$PSI={P}_{{\rm{adult}}}/{P}_{{\rm{leaf}}}$$

where *P*_adult_ and *P*_leaf_ are the relative phenophases of overwintering adults (the number of days after 1 March when AR1 or AR2 occurred) and plant leaves (the number of days after 1 March when LP2 or LP3 occurred) in 2014 or 2015. A *PSI* close to 1 indicates high degree of phenological synchrony between the adults and their host plants.

Based on the survival and fecundity of the groups of five paired adults, the following demographic parameters were estimated as described in a previous study^65^:

2 $${\rm{Gross}}\,{\rm{fecundity}}\,{\rm{rate}}=\sum _{x=\alpha }^{\beta }{M}_{x}$$

3 $${\rm{Net}}\,{\rm{fecundity}}\,{\rm{rate}}=\sum _{x=\alpha }^{\beta }{l}_{x}{M}_{x}$$

4 $${\rm{Daily}}\,{\rm{egg}}\,{\rm{production}}\,({\rm{eggs}}/{\rm{female}}/{\rm{day}})=\frac{{\sum }_{x=\alpha }^{\beta }{l}_{x}{M}_{x}}{{\sum }_{x=0}^{\omega }{l}_{x}}$$

where *x* is the age in days, *α* is the age at start of reproduction, *β* is the age at the end of reproduction, *ω* is the last possible day of life, *l*_*x*_ is the proportion of groups surviving to age *x*, and *M*_*x*_ is the average number of eggs laid by (living) females of age *x*.
